# Dr. Charles C. Dellenbaugh

**Published:** 1917-03

**Authors:** 


**Dr. Charles C. Dellenbaugh,** Buffalo 1859, died at his home in Portland, Mich., Dec. 25, aged 82.

